# The cognitive behavioural approach to the treatment of hallucinations. Is every experience of hallucination a part of the symptoms associated to psychosis - or even schizophrenia ?

**DOI:** 10.1192/j.eurpsy.2023.1029

**Published:** 2023-07-19

**Authors:** M. J. Goujon, E. Gallois

**Affiliations:** 1 Cellular Neurobiology Laboratory, Salk Institute for Biological Studies / UCSD, La Jolla, CA 92037, United States; 2 Unité d’Accueil Psychothérapeutique, Centre Hospitalier de l’Agglomération Montargeoise, Montargis, France

## Abstract

**Introduction:**

We report here our experience of treating hallucinations (auditory, visual, sensorial) using Cognitive Behavioural Therapy (CBT), along with medications. Our experience goes towards the conclusion that diagnosis is usually made prior to medical treatment of symptoms with high doses of neuroleptic drugs.

**Objectives:**

Our aim was to make a clear difference between hallucinations which need high doses of neuroleptics for cure and hallucinations that could respond to lesser drug treatment associated with CBT.

**Methods:**

Our method was based on individual sessions of CBT.

**Results:**

Our behavioural-cognitive method yielded high success rates as evidenced by thorough investigation into patients’ medical record including past medical history, prior drug use and life-threatening events.

This work is preliminary to a follow-up with the rigorous method for evaluation.

**Conclusions:**

Further, we aim to promote out-patient follow-ups in our unit after a very short inpatient assessment and treatment.

Our therapeutic approach is now approved by our team and new patients are currently being included.

**Disclosure of Interest:**

None Declared

